# A novel mutation of CTC1 leads to telomere shortening in a chinese family with interstitial lung disease

**DOI:** 10.1186/s41065-023-00299-4

**Published:** 2023-11-18

**Authors:** Lv Liu, Hua Luo, Yue Sheng, Xi Kang, Hong Peng, Hong Luo, Liang-Liang Fan

**Affiliations:** 1 Department of Pulmonary and Critical Care Medicine, Research Unit of Respiratory Disease, Hunan Diagnosis and Treatment Center of Respiratory Disease, the Second Xiangya Hospital, Central South University, Changsha, China; 2 Department of Cell biology, School of Life Science, Central South University, Changsha, China; 3 Department of Cardio-Thoracic Surgery, Changsha Medical School, the Affiliated Changsha Central Hospital, University of South China, Changsha, China

**Keywords:** Interstitial lung diseases, Pulmonary fibrosis, *CTC1* mutation, Short telomere length, Whole exome sequencing

## Abstract

Interstitial lung diseases (ILDs), or diffuse pulmonary lung disease, are a subset of lung diseases that primarily affect lung alveoli and the space around interstitial tissue and bronchioles. It clinically manifests as progressive dyspnea, and patients often exhibit a varied decrease in pulmonary diffusion function. Recently, variants in telomere biology-related genes have been identified as genetic lesions of ILDs. Here, we enrolled 82 patients with interstitial pneumonia from 2017 to 2021 in our hospital to explore the candidate gene mutations of these patients via whole-exome sequencing. After data filtering, a novel heterozygous mutation (NM_025099: p.Gly131Arg) of *CTC1* was identified in two affected family members. As a component of CST (CTC1-STN1-TEN1) complex, CTC1 is responsible for maintaining telomeric structure integrity and has also been identified as a candidate gene for IPF, a special kind of chronic ILD with insidious onset. Simultaneously, real-time PCR revealed that two affected family members presented with short telomere lengths, which further confirmed the effect of the mutation in the *CTC1* gene. Our study not only expanded the mutation spectrum of *CTC1* and provided epidemiological data on ILDs caused by *CTC1* mutations but also further confirmed the relationship between heterozygous mutations in *CTC1* and ILDs, which may further contribute to understanding the mechanisms underlying ILDs.

## Introduction

CTC1-STN1-TEN1 (CST) is an RPA-like complex that binds single-stranded DNA with high affinity and plays a crucial role in telomere maintenance in different ways [1, 2]. For example, CST can facilitate efficient replication of telomeric DNA and prevent catastrophic telomere loss [3]. CST can also participate in the late S/G2-specific synthesis of telomeric C-strands, and the depletion of CST can lead to excessively long G-overhangs [4]. In addition, CST may compete with the shelterin subunits POT1-TPP1 for binding to telomeric DNA and restrict telomerase extension of telomeres [5].

As a component of CST, conserved telomere maintenance component 1 (CTC1) is responsible for protecting telomeres from degradation and forming the alpha-accessory factor complex together with STN1 [6, 7]. The *CTC1* gene is located on chromosome 17p13.1, and it consists of 23 exons, spanning approximately 23.2 kilobases. In 2012, Anderson et al. described that variants of CTC1 were responsible for an autosomal recessive pleomorphic disorder named Coats plus syndrome, which featured intracranial calcifications, leukodystrophy, brain cysts, retinal telangiectasia and exudate extraneurologic manifestations [8]. Since then, a spectrum of phenotypes, including bone marrow failure, colorectal cancer and dyskeratosis congenita, have also been detected in patients with *CTC1* mutations [9–11]. In 2018, Deng et al. first found a heterozygous mutation of *CTC1* in a Chinese sporadic idiopathic pulmonary fibrosis (IPF) patient [12]. In 2019, Arias-Salgado et al. further confirmed that heterozygous mutation of *CTC1* was the genetic lesion of IPF [13]. Hence, *CTC1* is considered a telomere biology-related gene that is responsible for different telomere biology disorders. including interstitial lung diseases (ILDs) and dyskeratosis congenita [14].

ILDs are a subset of disorders that mainly affect alveoli and the area around interstitial tissue and bronchioles [15]. It clinically manifests as progressive dyspnea, and patients often exhibit a varied decrease in pulmonary diffusion function [15]. The abovementioned IPF is a special kind of chronic ILD with insidious onset [15]. Here, whole-exome sequencing was employed to explore the genetic lesions of 82 unrelated patients with ILDs, and a novel heterozygous mutation (NM_025099: p.Gly131Arg) of *CTC1* was identified in a patient with IPF. Bioinformatics software predicted that the novel mutation of *CTC1* was deleterious. Real-time PCR revealed that the length of telomeres in the mutation carriers was also shorter than that in the healthy controls.

## Materials and methods

### Subjects

**Fig. 1:**
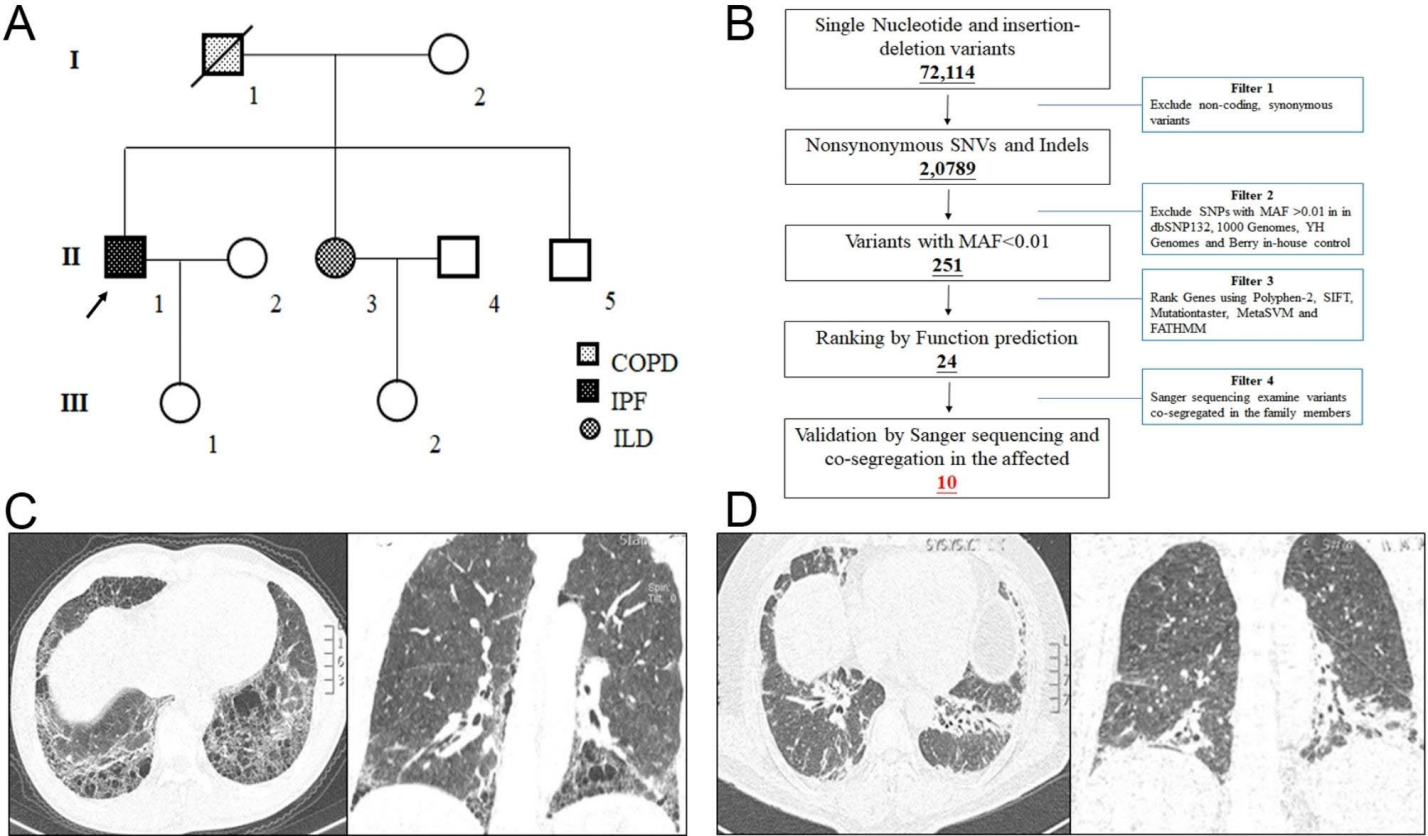
The clinical data of the family. (**A**) Pedigree of the family. White circles/squares are unaffected, arrow indicates the proband. (**B**) Schematic representation of the filter strategies employed in our study. The high-resolution CT of the proband (II-1) (**C**) and II-3 (**D**)

In total, 82 unrelated patients who were diagnosed with ILDs or related interstitial lung disease at the Second Xiangya Hospital participated in the study. In this reported family, nine family members were investigated, and blood was obtained from eight family members, including two affected individuals (Fig. 1A). The affected members were reviewed with high-resolution computed tomography (CT).

### Whole-exome sequencing and Sanger sequencing

Genomic DNA was isolated from peripheral blood lymphocytes of all the patients with a DNeasy Blood & Tissue Kit (Qiagen 69504) following the manufacturer’s instructions. The proband (II-1) was selected to perform the whole sequencing. Whole-exome sequencing and regular filtering analysis were conducted by BerryGenomics Biotech Company (Beijing, China) as we previously described [16]. The strategies of data filtering are shown in Fig. 1B. Polymerase chain reaction (PCR) with designed primers (Forward 5’-3’ GGACCTCAAGACTCACCAGC, Reserve 5’-3’ AGCATCCTATCCACCCACCT) was performed in a Mastercycler® X50 PCR machine (Eppendorf, Germany), and the products were sequenced by an ABI 3100 Genetic Analyzer (ABI, USA).

### Functional study

The structure of the CTC1 protein was built by Swiss-Model software (https://swissmodel.expasy.org/interactive), and the local hydrophobicity was analyzed by ProtScale based on the structure as we previously described [17].

A total of 60 ng DNA was prepared for each real-time PCR system and treated with a telomere length assay kit (Biowing Telomere Detection Kit including 1500 random peripheral blood samples data from Shaihai, Shanghai Biowing Applied Biotechnology Co., Ltd) according to established protocols [18]. The Fast 7500 Real-Time PCR Systems (Applied Biosystems) and 2^(−△△Ct)^ methods were used to compare the telomere length of each group. Sample collected and run independently of each other.

## Results

### Clinical description

The family came from Hunan Province, China. Proband 1 (II-1), a 56-year-old male, was admitted to our hospital due to cough and postexercise dyspnea for 1 year. He denied smoking and occupational exposure. The antibody test for connective tissue diseases showed a slight increase in rheumatoid factor antibodies (IgM and IgA). A lung function test showed mild obstructive dysfunction of pulmonary ventilation. High-resolution CT presented bilateral lower predominate subpleural honeycomb shadows, which were in accordance with the UIP pattern (Fig. 1C). The patient was clinically diagnosed with IPF and referred to two professional radiologists and one respiratory specialist. However, the patient refused to receive further bronchoscopy tests and medical treatment with pirfenidone and was clinically stable through further telephone follow-up. A family history survey found that his father died from chronic obstructive pulmonary disease, his sister (II-3) claimed shortness of breath after general activities, and high-resolution CT showed obvious ground glass shadows (Fig. 1D).

### Genetic analysis

**Fig. 2:**
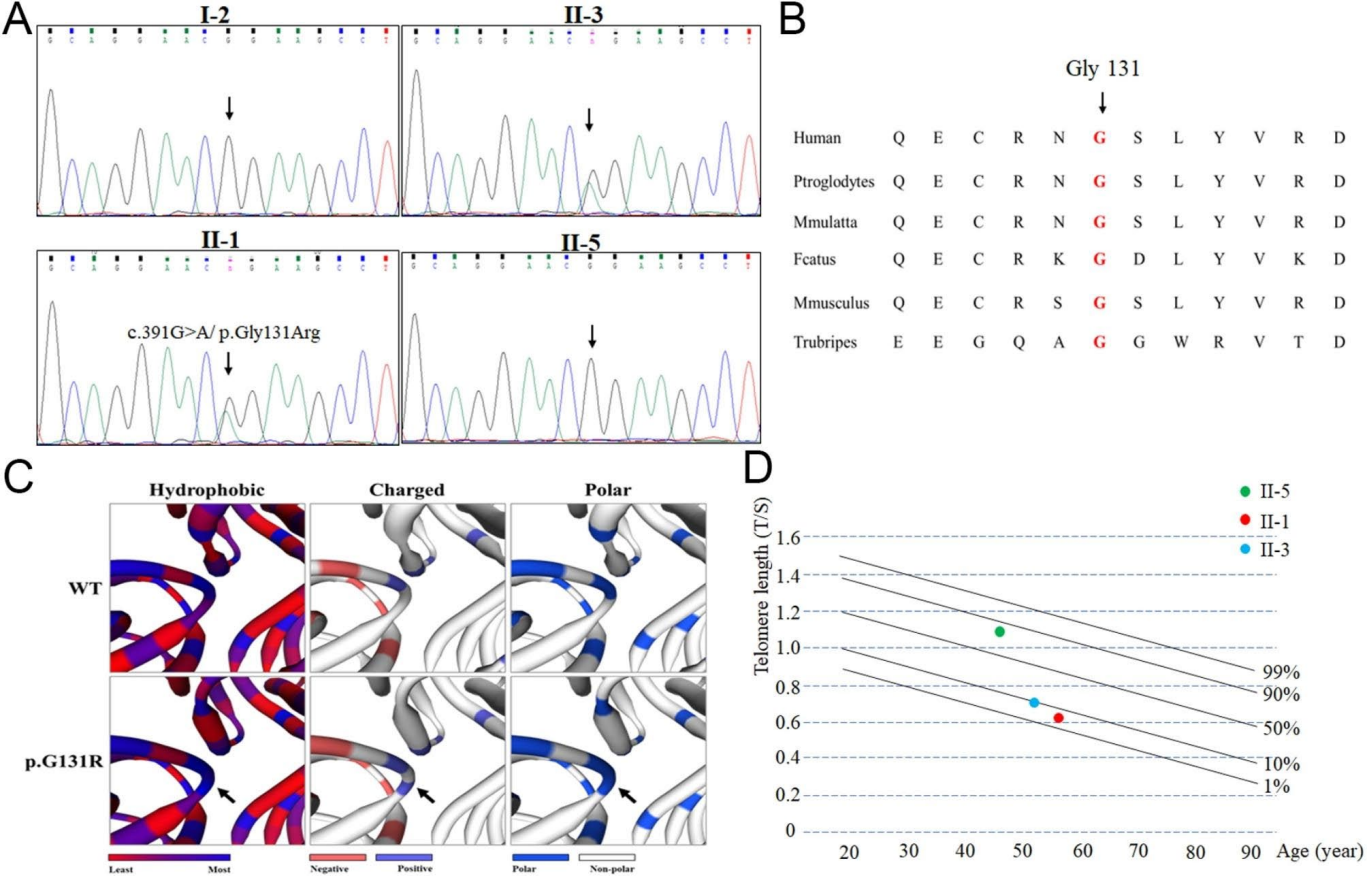
The genetic analysis of the family. (**A**) Sanger DNA sequencing chromatogram demonstrates the heterozygosity for a CTC1 missense mutation (c.391G > A/ p.Gly131Arg) in the family. (**B**) Alignment of multiple CTC1 protein sequences across species. The Gly131 affected amino acid locates in the highly conserved amino acid region in different mammals (from Ensembl). Red column shows the Gly131 site. (**C**) The wild type CTC1 (WT) protein structure and the mutant CTC1 (p.G131R) protein structure were predicted by SWISS-MODEL online software. The hydrophobic surface area, surface charge and polarity of the WT and mutated CTC1 were predicted. (**D**) Telomere length of the mutation carriers (II-1 and II-3) and healthy family member (II-5).

Whole-exome sequencing was applied to analyze the candidate gene for the proband. After alignment and single nucleotide variant calling, 72,114 variants were identified in the proband. Via the abovementioned filtering method (Fig. 1B) and Sanger sequencing validation, 10 mutations remained (Table 1). Among these 10 mutations, only the novel mutation (NM_025099: c.391G > A/ p.Gly131Arg) of *CTC1* could serve as the underlying genetic lesion for the family (Fig. 2A). The novel mutation, resulting in a substitution of glycine by arginine, was located in a highly evolutionarily conserved site (Fig. 2B). Structural analysis further revealed that the p.Gly131Arg mutation changed the hydrophobic surface area, surface charge and polarity of the CTC1 protein (Fig. 2C). To confirm the effects of the novel mutation, two mutation carriers of the family were enrolled to detect the telomere length by real-time PCR. The results showed that the telomere length of the two affected patients (II-1 and II-3) was shorter than that of healthy controls and one of the relatives without the CTC1 variant (II-5) (Fig. 2D), which indicated that mutations in *CTC1*, a telomere biology-related gene [14], may reduce the length of telomeres and lead to IPF and related diseases.

**Table 1:** The mutation list of Sanger sequencing validation and co-segregation analysis

Chr	POS	RB	AB	Gene	Transcript	SIFT	Polyphen-2	Mutationtaster
2	74,466,475	T	C	SLC4A5	NM_021196.3:p.Tyr769Cys/c.2306 A > G	0 (D)	0.993(D)	1 (D)
4	8,454,607	A	G	TRMT44	NM_152544.2:c.1024-2 A > G	-	-	1 (D)
5	52,979,034	A	G	NDUFS4	NM_001282136.2:p.Pro134Arg/c.401 C > G	0 (D)	0.980 (D)	1 (D)
8	125,115,444	G	A	FER1L6	NM_001039112.2:p.Cys1728Tyr/c.5183G > A	0 (D)	0.999 (D)	1 (D)
9	2,718,935	C	T	KCNV2	NM_133497.3:p.Ala399Val/c.1196 C > T	0 (D)	1 (D)	1 (D)
15	50,288,937	G	A	ATP8B4	NM_024837.3:p.Arg176Cys/c.526 C > T	0 (D)	0.989 (D)	0.999 (D)
16	72,020,218	G	A	PKD1L3	NM_181536.1:p.Gln246*/c.736 C > T	0 (D)	1 (D)	1 (D)
17	8,141,754	C	T	CTC1	NM_025099.5:p.Gly131Arg/c.391G > A	0 (D)	1 (D)	0.999 (D)
17	47,241,527	A	G	B4GALNT2	NM_153446.2:p.Ser342Gly/c.1024 A > G	0 (D)	0.986 (D)	0.999 (D)
19	6,444,216	C	T	SLC25A23	NM_024103.2:p.Gly390Ser/c.1168G > A	0 (D)	0.90 (D)	1 (D)

Chr, Chromosome; POS, position; RB, reference sequence base; AB, alternative base identified; D, deleterious

## Discussion

It is widely accepted that both genetic factors and environmental elements are involved in the occurrence and development of ILDs [19]. Mutations in telomere biology-related genes and surfactant protein-related genes are two major genetic lesions of ILDs [19]. At present, more than ten telomere biology-related genes have been identified in patients with ILDs, such as *dyskerin* (*DKC1*), *regulator of telomere elongation helicase 1*, (*RTEL1*) *NHP2 ribonucleoprotein* (*NHP2*) and *NOP10 ribonucleoprotein* (*NOP10*) [12, 20]. As a component of CST complex, CTC1 is responsible for maintaining telomeric structure integrity and has also been identified as a candidate gene for IPF [12, 13, 21]. At present, a total of 51 mutations have been reported in patients, and most of them were identified in Coats plus syndrome patients or cerebroretinal microangiopathy with calcifications and cysts. Only four variants have been detected in patients with ILDs [8, 12, 13]. Here, we identified a novel mutation (NM_025099: p.Gly131Arg) in *CTC1* in a family with IPF. Our study may expand the mutation spectrum of *CTC1* and further prove that mutations in *CTC1* may lead to ILDs.

The CTC1 protein contains four oligonucleotide/oligosaccharide-binding (OB)-fold domains, which are responsible for forming CST complex by binding to STN1-TEN1 [22, 23]. Previous in vitro assays suggested that mutations in OB-fold domain may affect full-length CTC1 localization to telomeres and STN1-TEN1 binding [22]. In this study, p.Gly131Arg was located in the OB-fold domain of CTC1. Bioinformatics analysis indicated that the mutation may change the hydrophobic surface area, surface charge and polarity of CTC1, which may further disrupt the structure and function of CST complex [23].

*CTC1* mutations were first identified in Coat Plus and dyskeratosis congenita [8, 24], two types of autosomal recessive disorders that are associated with telomere maintenance defects. In 2012, Anderson et al. first identified compound heterozygous variation (c.724_727delAAAG and c.2611G > A) of *CTC1* in a young female who died from pulmonary fibrosis at the age of 28 and presented with dystrophic nails, thin hair, fractures, anemia, and gastrointestinal ectasia [8]. Until 2018, heterozygous mutation of *CTC1* was validated in patients with pulmonary fibrosis [12]. At present, only two studies have reported that heterozygous mutation of *CTC1* was the genetic lesion of pulmonary fibrosis patients [12, 13]. In this study, we identified a novel heterozygous mutation of *CTC1* in a family with IPF and ILD, which further supports previous findings of heterozygous *CTC1* mutations in patients with IPF.

Short telomere lengths have been identified in all kinds of ILDs and have been associated with poorer survival for IPF patients [25]. Mutations in several telomere biology-related genes have been identified in patients with ILDs [20]. However, the relationship between the underlying pathogenesis of ILDs and the genes involved in telomere-related components, telomere maintenance, and telomerase activity is still not clear. In the Coat plus study, biallelic *CTC1* mutation carriers showed relatively shorter telomere lengths than heterozygous mutation carriers [8]. In an IPF study, a 51-year-old male pulmonary fibrosis patient with a heterozygous CTC1 mutation presented extremely shortened telomeres [13]. In this study, we also found that two mutation carriers presented with short telomere length compared to healthy control and one non-affected relative. This study further confirmed that mutations in *CTC1* may lead to short telomere length and result in different diseases, including ILDs. The occurrence of different kinds of diseases caused by *CTC1* mutations may be due to genetic heterogeneity, which is similar to other telomere biology-related genes, such as *DKC1* and *RTEL1* [26, 27].

In summary, we identified a novel heterozygous mutation (NM_025099: p.Gly131Arg) in *CTC1* from one out of 82 ILD patients. Two mutation carriers in this family presented with short telomere length compared to healthy controls. Our study may expand the mutation spectrum of *CTC1* and provide epidemiological data on ILDs caused by *CTC1* mutations. We also further confirmed the relationship between heterozygous mutation of *CTC1* and ILDs, which may further contribute to understanding the mechanisms underlying ILDs.

## Data Availability

All supporting data of this article are included in the submitted manuscript.
